# Transient Dietary Intervention Induces Healthy Adipose Tissue Expansion and Metabolically Healthy Obesity in Mice

**DOI:** 10.1096/fj.202501121R

**Published:** 2025-07-16

**Authors:** Eri Wada, Hirotaka Hosono, Miyako Tanaka, Fumi Miyakawa, Kozue Ochi, Hiro Kohda, Shogo Tanno, Reon Shimano, Ayaka Ito, Yasuyuki Kitaura, Kazuya Ichihara, Akinobu Matsumoto, Tomoo Ogi, Noriko Satoh‐Asahara, Toyoaki Murohara, Takayoshi Suganami

**Affiliations:** ^1^ Department of Molecular Medicine and Metabolism, Research Institute of Environmental Medicine Nagoya University Nagoya Japan; ^2^ Department of Cardiology Nagoya University Graduate School of Medicine Nagoya Japan; ^3^ Department of Immunometabolism Nagoya University Graduate School of Medicine Nagoya Japan; ^4^ Department of Applied Biosciences, Graduate School of Bioagricultural Sciences Nagoya University Nagoya Japan; ^5^ Institute for Advanced Research Nagoya University Nagoya Japan; ^6^ Department of Food and Nutritional Sciences, College of Bioscience and Biotechnology Chubu University Kasugai Japan; ^7^ Division of Biological Science, Graduate School of Science Nagoya University Nagoya Japan; ^8^ Department of Genetics, Research Institute of Environmental Medicine Nagoya University Nagoya Japan; ^9^ Department of Endocrinology, Metabolism, and Hypertension Research Clinical Research Institute, NHO Kyoto Medical Center Kyoto Japan; ^10^ Institute of Nano‐Life‐Systems, Institutes of Innovation for Future Society Nagoya University Nagoya Japan; ^11^ Center for One Medicine Innovative Translational Research (COMIT) Nagoya University Nagoya Japan; ^12^ Research Institute for Quantum and Chemical Innovation, Institutes of Innovation for Future Society Nagoya University Nagoya Japan; ^13^ Innovative Research Center for Preventive Medical Engineering, Institutes of Innovation for Future Society Nagoya University Nagoya Japan

**Keywords:** chronic inflammation, fibroblast, fibrosis, ketone body, obesity, weight‐cycling

## Abstract

As obesity progresses, dynamic tissue remodeling of adipose tissue occurs over time, that is, adipocyte hypertrophy, chronic inflammation, and interstitial fibrosis. Some obese individuals exhibit healthy adipose tissue expansion, characterized by modest inflammation and fibrosis despite adipocyte hypertrophy, resulting in “Metabolically Healthy Obesity (MHO)”. In this study, we investigated the effects of transient weight loss on adipose tissue remodeling during the development of obesity. Male C57BL6/J mice received various types of transient weight loss treatments during diet‐induced obesity. A 2‐week weight loss intervention during the inflammatory phase promoted healthy adipose tissue expansion, reduced ectopic lipid accumulation, and improved glucose metabolism. In contrast, protocols with shorter duration and delayed intervention, failed to induce MHO. Since serum concentrations of ketone bodies were elevated during weight loss, we examined the effects of hyperketonemia on obesity‐induced adipose tissue remodeling. Transient treatment with 1,3‐butanediol (BD), which increased serum ketone body concentrations to levels similar to those observed during weight loss, induced healthy adipose tissue expansion and reduced hepatic steatosis even during continuous high‐fat diet (HFD) feeding. Ketone bodies effectively suppressed activation of adipose tissue fibroblasts in vivo and in vitro. This study provides evidence that an appropriate dietary intervention can promote healthy adipose tissue expansion in mice, even after the regaining of weight, thereby leading to MHO. As the underlying mechanism, our data revealed a key role for ketone bodies in suppressing activation of adipose tissue fibroblasts. This study paves the way for nutritional approaches to induce MHO.

## Graphical abstract

This study demonstrates that an appropriate dietary intervention can promote healthy adipose tissue expansion in mice, even after weight regain, leading to metabolically healthy obesity (MHO), whereas inappropriate protocols, such as those with short duration and late initiation, failed to do so. Regarding the underlying mechanism, our data indicate a key role of hyperketonemia during weight loss, which ameliorates adipose tissue fibrosis. Beyond genetic engineering and drug treatment, this study paves the way for nutritional approaches to induce MHO.

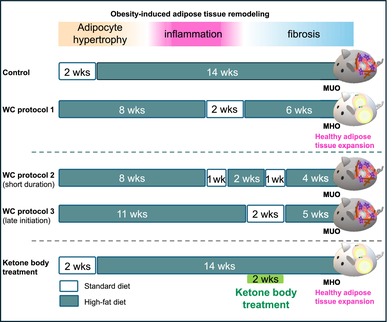

**Abbreviations**
AU: arbitrary unit
BD: 1,3‐butanediol
CLSs: crown‐like structures
HFD: high‐fat diet
MHO: metabolically healthy obesity
MUO: metabolically unhealthy obesity
PDGFRα: platelet‐derived growth factor receptor‐α
PPARγ: peroxisome proliferator‐activated receptor γ
RNA‐seq: RNA‐sequencing
SD: standard diet
SVF: stromal vascular fraction
TGFβ: transforming growth factor‐β
WC: weight‐cycling
WL: weight loss
αSMA: α‐smooth muscle actin
βHB: β‐hydroxybutyrate

## 1 Introduction

Obesity is a condition characterized by excessive energy storage in adipose tissue, causing lifestyle‐related diseases. Not all individuals with obesity develop complications, and clear individual differences exist. Thus, the concepts of “Metabolically Healthy Obesity (MHO)”, where individuals are healthy despite being obese, and “Metabolically Unhealthy Obesity (MUO)”, which is accompanied by various metabolic disorders, have been proposed [1]. The features of MUO include chronic inflammation in adipose tissue and ectopic lipid accumulation in the liver and skeletal muscle [2, 3]. In adipose tissue, chronic overnutrition induces adipocyte hypertrophy, followed by immune cell infiltration, angiogenesis, and overproduction of extracellular matrix. Such dynamic remodeling of adipose tissue underlies metabolic derangements such as hepatic steatosis [4]. In particular, substantial attention has been given to unique histological structures termed crown‐like structures (CLSs), where infiltrating macrophages surround and engulf adipocytes that have undergone cell death due to metabolic stress [4]. CLSs serve as a driving force not only for chronic inflammation but also for interstitial fibrosis in adipose tissue, where some adipocyte progenitor cells transdifferentiate into myofibroblasts, acquiring profibrotic properties [5, 6, 7]. In contrast, adipose tissue in MHO does not exhibit chronic inflammation and fibrosis, and is referred to as “healthy adipose tissue expansion.” However, in actual clinical practice, weight loss treatment for obesity is primarily evaluated using body weight and body fat mass as indices, with minimal considerations given to the aforementioned concepts of MHO and MUO.

Dietary intervention is a fundamental treatment for obesity that is applied to all patients, and, when successful, it leads to a reduction in the risk of various complications. However, maintaining appropriate dietary interventions in the long term is challenging, and weight regain often occurs, a phenomenon known as weight‐cycling (WC) [8]. The effects of WC have been debated, with some human studies reporting that WC increases the risk of metabolic syndrome, while other reports indicate no increased risk compared to those who remain overweight [9, 10, 11, 12, 13]. Similarly, animal experiments using various dietary protocols have shown conflicting results, and their effects on systemic metabolism remain inconsistent [14, 15, 16]. It has been reported that WC affects a variety of stromal cells, including immune cells, in adipose tissue; both proinflammatory and anti‐inflammatory responses are associated with weight loss [17, 18, 19, 20, 21]. The effects of transient weight loss on adipose tissue remodeling during WC remain to be elucidated.

We have previously reported on the molecular mechanisms underlying obesity‐induced adipose tissue inflammation, focusing on cell‐to‐cell interactions between parenchymal adipocytes and stromal cells [22, 23, 24]. In particular, we have elucidated the pathophysiologic role of macrophage‐inducible C‐type lectin (Mincle) in obesity‐induced adipose tissue fibrosis, which originates from CLSs [25, 26]. Although Mincle is known as a pathogen sensor against *Mycobacterium tuberculosis* and pathogenic fungi, we found that it is selectively expressed in macrophages within CLSs, where Mincle senses adipocyte death, inducing myofibroblast accumulation and accelerating interstitial fibrosis [25, 26]. Namely, Mincle deficiency suppresses obesity‐induced adipose tissue fibrosis, resulting in enlarged adipocytes and reduced hepatic steatosis [25]. We have also reported that a sodium glucose co‐transporter 2 inhibitor effectively promotes healthy adipose tissue expansion in diet‐induced obese mice [27]. A similar phenotype has been observed in the absence of type VI collagen, which is characteristic of adipose tissue [28]. These findings, taken together, indicate that adipose tissue fibrosis plays an essential role in the pathophysiology of healthy adipose tissue expansion. However, dietary approaches to promote healthy adipose tissue expansion during WC have not been explored.

In this study, we tested various WC protocols in a mouse model of diet‐induced obesity and found that an appropriate dietary intervention can promote healthy adipose tissue expansion. We observed an improvement in adipose tissue fibrosis during weight loss, characterized by reduced myofibroblast accumulation and decreased collagen deposition. Mechanistically, the transient administration of ketone bodies during the weight loss period could mimic the effects of the WC protocol. These findings may contribute to the development of novel nutritional interventions aimed at inducing MHO.

## 2 Materials and Methods

### 2.1 Reagents

All reagents were purchased from Sigma‐Aldrich (St Louis, MO) or Nacalai Tesque (Kyoto, Japan) unless otherwise noted. Antibodies and primers used in this study are listed in Tables S1 and S2, respectively.

### 2.2 Animals

C57BL/6J wild‐type male mice were purchased from CLEA Japan (Tokyo, Japan). They were maintained in a temperature‐, humidity‐, and light‐controlled room (12 h light/dark cycles), allowed free access to water and standard diet (SD; CE‐2; 343.1 kcal per 100 g, 12.6% energy as fat; CLEA Japan) or high‐fat diet (HFD; D12492: kcal per 100 g, 60% energy as fat; Research diet, New Brunswick, NJ). To examine the effect of ketone bodies, mice were given water containing 20% of 1,3‐butanediol (BD) *ad libitum*. For the glucose tolerance test, mice were intraperitoneally injected with glucose (1.5 g/kg body weight) after 16 h of fasting. For the insulin tolerance test, randomly fed mice were intraperitoneally injected with 0.75 U/kg insulin (Humulin R; Eli Lilly, Indianapolis, IN). All animal experiments, approved by the Committee on the Ethics of Animal Experiments of Nagoya University (approval number 240021), were carried out according to the ARRIVE guidelines.

### 2.3 Blood and Liver Analyses

Blood analyses were performed as described previously [29]. Blood β‐hydroxy butyrate (βHB) was measured by Stat strip XP3 (NIPRO, Osaka, Japan). Serum concentrations of insulin (Morinaga Institute of Biological Science, Kanagawa, Japan) and adiponectin (Otsuka Pharmaceutical Co. Ltd., Tokyo, Japan) were determined using ELISA kits. The lipids in the liver were analyzed as described previously [25].

### 2.4 Histological Analysis

Liver and epididymal adipose tissue were fixed with 10% neutral‐buffered formalin and embedded in paraffin. Two‐μm‐thick sections were stained with hematoxylin and eosin, and 4‐μm‐thick sections were stained with Sirius red. For the measurement of adipocyte cell size, more than 200 cells were counted in each section using image analysis software (Dynamic cell count; KEYENCE, Osaka, Japan). Immunohistochemical staining was performed as previously described [25]. All samples were analyzed with a fluorescence microscope (BZ‐X710; KEYENCE). The quantitative histological analysis was performed by two investigators who had no knowledge of the origin of the slides.

### 2.5 RNA‐Sequencing Analysis

**FIGURE 1:**
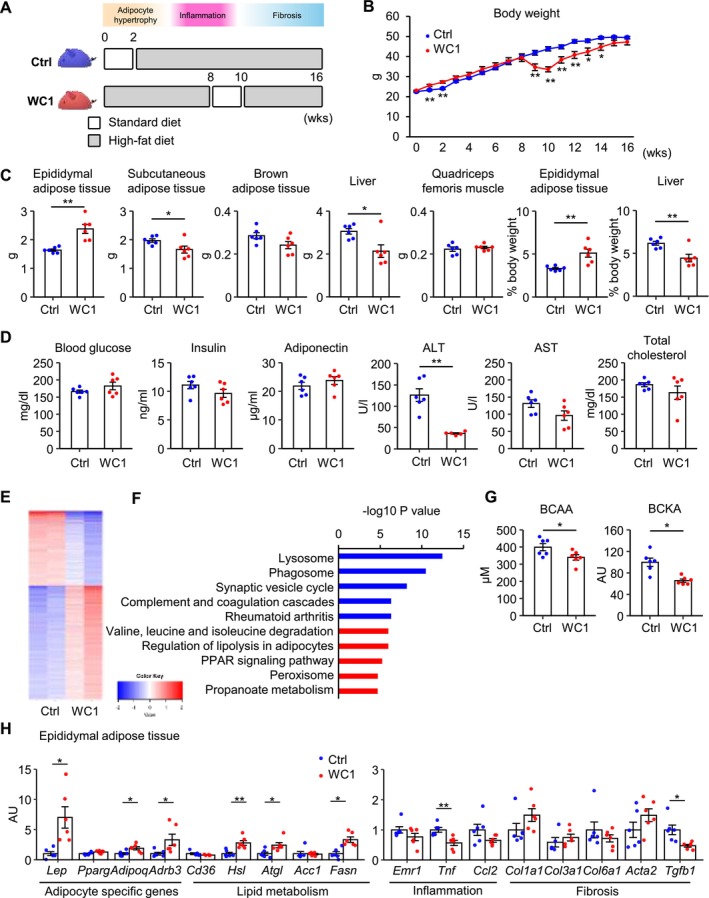
Diet‐induced weight‐cycling induces metabolically healthy obesity in mice. (A) Schematic diagram of weight‐cycling model 1 (WC1) and its control (Ctrl). The WC1 mice were fed high‐fat diet (HFD) for 8 weeks, followed by standard diet (SD) for 2 weeks for weight loss, then HFD again for additional 6 weeks, whereas the Ctrl mice were fed HFD continuously for 14 weeks. *n* = 6 per group. (B) Body weight curves. (C) Tissue weights and the percentages of tissue weight relative to body weight. (D) Blood glucose levels and serum parameters. (E, F) RNA‐sequencing analysis of the epididymal adipose tissue. *n* = 2 per group. (E) Clustering analysis (*k*) and (F) Kyoto Encyclopedia of Genes and Genomes (KEGG) enrichment analysis (*k* = 2). (G) Serum concentrations of branched‐chain amino acids (BCAA) and branched‐chain α‐keto acids (BCKA). (H) mRNA expression levels in epididymal adipose tissue. Data are expressed as means ± SEM. *p < 0.05 and **p < 0.01 by Student's *t* test.

Total RNA extracted from epididymal adipose tissue samples was purified using an RNeasy MiniElute Cleanup Kit (QIAGEN, Hilden, Germany). For the WC model 1 (WC1) experiment (Figure 1), RNA‐sequencing (seq) analysis was performed as previously described [30]. For the weight loss experiment (Figure 4), RNA‐seq analysis was performed by Novogene Co. Ltd. (Beijing, China). Genes expressed below 0.5 counts per million were filtered out from analysis. Fold changes of gene expression levels were analyzed using integrated Differential Expression & Pathway (iDEP) v.0.96 [31] and Gene set enrichment analysis (GSEA) [32, 33]. The RNA‐seq data are available on the website of the Gene Expression Omnibus at the National Center for Biotechnology Information (GSE286420 and GSE286421).

### 2.6 Quantitative Real‐Time PCR

Quantitative real‐time PCR was performed as previously described [29]. Data were normalized to 36B4 levels and analyzed using the comparative CT method.

### 2.7 Preparation of Primary Fibroblasts

The stromal vascular fraction (SVF) derived from epididymal adipose tissue was obtained as previously described [25]. CD45(−) platelet‐derived growth factor receptor‐α (PDGFRα)(+) fibroblasts in the SVF were sorted using an SH800 cell sorter (SONY, Tokyo, Japan), and CD45(−), CD31(−), and Ter119(−) cells were obtained using a magnetic cell sorting system (AutoMACS; Miltenyi Biotec, Bergisch Gladbach, Germany). Macrophages were identified in the SVF as CD45(+) and F4/80(+) cells. The fibroblasts were maintained in Dulbecco's Modified Eagle Medium (DMEM) supplemented with 10% fetal bovine serum and antibiotics. The cells were deprived of serum and glucose and treated with 5 mM βHB overnight, followed by 1 ng/mL transforming growth factor‐β (TGFβ) for 24 h.

### 2.8 Immunoblot Analysis

Total cell lysates from the epididymal adipose tissue were prepared as described previously [25]. Immunoblots were analyzed using a LuminoGraph chemiluminescence imaging system (ATTO, Tokyo, Japan).

### 2.9 Metabolomic Analysis

The hydroxyproline content in the mouse epididymal adipose tissue was measured by Human Metabolome Technologies (Tsuruoka, Japan). Serum amino acids and βHB were measured by gas chromatography/mass spectrometry as previously described [34].

### 2.10 Statistical Analysis

Data are means ± SEM, and *p* < 0.05 was considered statistically significant. Statistical analysis was performed using analysis of variance (ANOVA), followed by a Tukey–Kramer test. An unpaired *t* test was used to compare two groups.

## 3 Results

### 3.1 Diet‐Induced WC Induces MHO in Mice

In this study, we employed HFD‐induced obese mice on the C57BL/6J background. The mice sequentially exhibited adipocyte hypertrophy, immune cell infiltration (inflammatory phase), and interstitial fibrosis (fibrotic phase) (Figure S1) as reported previously [24, 25]. We fed HFD or SD to 8‐week‐old male C57BL/6J mice to establish a WC1 protocol. The WC1 group was fed HFD for 8 weeks, followed by SD for 2 weeks for weight loss, then HFD again for an additional 6 weeks. The control group was fed HFD continuously for 14 weeks from 10 weeks of age (Figure 1A,B). At the end of the experimental period, there was no significant difference in body weight between the groups (Figure 1B). The weight of epididymal adipose tissue was significantly increased, whereas the weights of the liver and subcutaneous adipose tissue were significantly decreased in WC1 mice compared to control mice (Figure 1C), indicating a shift in lipid distribution within the body. The serum concentrations of alanine aminotransferase (ALT) significantly decreased and insulin tended to decrease, while those of adiponectin tended to increase in WC1 mice (Figure 1D).

At the end of the experimental period, we investigated gene expression profiles in epididymal adipose tissue, where chronic inflammation primarily occurs. Pathway analysis of downregulated gene sets in WC1 mice highlighted pathways such as “Lysosome” and “Phagocytosis,” whereas analysis of upregulated gene sets underlined “Valine, leucine, and isoleucine degradation,”, “Regulation of lipolysis in adipocytes,” and “Peroxisome proliferator‐activated receptor (PPAR) signaling pathway” (Figure 1E,F). These observations were consistent with the notion that WC1 mice exhibit healthy adipose tissue expansion. Indeed, the serum concentrations of valine, leucine, and isoleucine, referred to as branched‐chain amino acids (BCAA), along with their metabolites such as branched‐chain keto acids (BCKA), were decreased in WC1 mice compared to control mice (Figure 1G). Recent evidence has shown that BCAA metabolism is impaired in mice and humans with obesity, leading to increased serum BCAA and BCKA concentrations [35]. We also examined the gene expression profiles using quantitative PCR (Figure 1H). The expression of adipocyte‐specific genes, such as adiponectin (*Adipoq*), and lipolytic genes, such as hormone‐sensitive lipase (*Hsl*) and adipose triglyceride lipase (*Atgl*), was significantly increased, while the expression of inflammation‐ and fibrosis‐related genes, such as tumor necrosis factor‐α (*Tnf*) and *Tgfb1*, respectively, was decreased in WC1 mice. On the other hand, subcutaneous and brown adipose tissues showed only slight changes in gene expression (Figure S2A,B).

### 3.2 WC Leads to Healthy Adipose Tissue Expansion and Reduced Hepatic Steatosis

**FIGURE 2:**
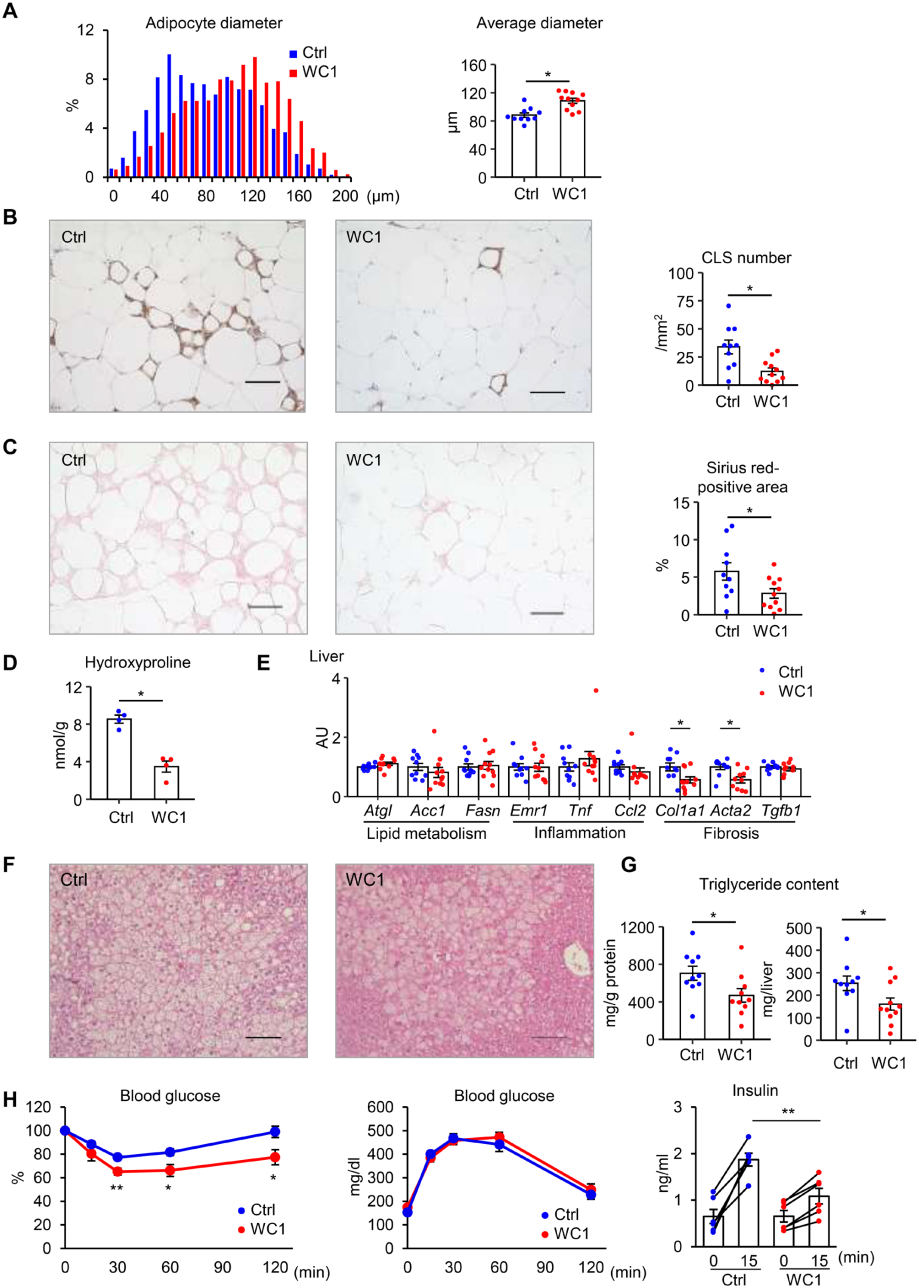
WC leads to healthy adipose tissue expansion and reduced hepatic steatosis. (A) Histogram of adipocyte diameters in epididymal adipose tissue from WC1 and Ctrl mice. *n* = 10–11. (B) Representative images of F4/80 immunostaining and the number of crown‐like structures (CLSs) in epididymal adipose tissue. (C) Representative images of Sirius red staining and quantification of the Sirius red–positive area in epididymal adipose tissue. (D) Hydroxyproline content in epididymal adipose tissue. *n* = 4 per group. (E) mRNA expression levels in liver. (F) Representative images of hematoxylin and eosin staining in liver. (G) Hepatic triglyceride content. (H) Insulin tolerance test (*lt*.) and glucose tolerance test (*rt*.). Serum insulin concentrations were measured before and 15 min after glucose administration during the glucose tolerance test. *n* = 6 per group. Data are expressed as means ± SEM. *p < 0.05 and **p < 0.01 by Student's *t* test. Scale bars, 100 μm.

Next, we assessed histological changes of epididymal adipose tissue and found that adipocyte size was significantly increased in WC1 mice compared to control mice (Figure 2A). The number of CLSs, as evaluated by F4/80 immunostaining, was significantly decreased in WC1 mice (Figure 2B). Sirius red staining, along with hydroxyproline content, revealed a significant reduction of adipose tissue fibrosis in WC1 mice (Figure 2C,D). These histological features are consistent with gene expression profiles related to inflammation and fibrosis (Figure 1H). Although there were no apparent changes in the expression of lipidmetabolism‐related genes in the liver (Figure 2E), histological analysis and triglyceride measurements revealed reduced hepatic steatosis in WC1 mice (Figure 2F,G). Moreover, WC1 mice showed better insulin sensitivity than control mice (Figure 2H). Collectively, these findings suggest that the WC1 protocol induces healthy adipose tissue expansion and reduced ectopic lipid accumulation in the liver, thereby exhibiting the MHO‐like phenotype.

### 3.3 Other WC Protocols Show Differential Effects on Adipose Tissue

**FIGURE 3:**
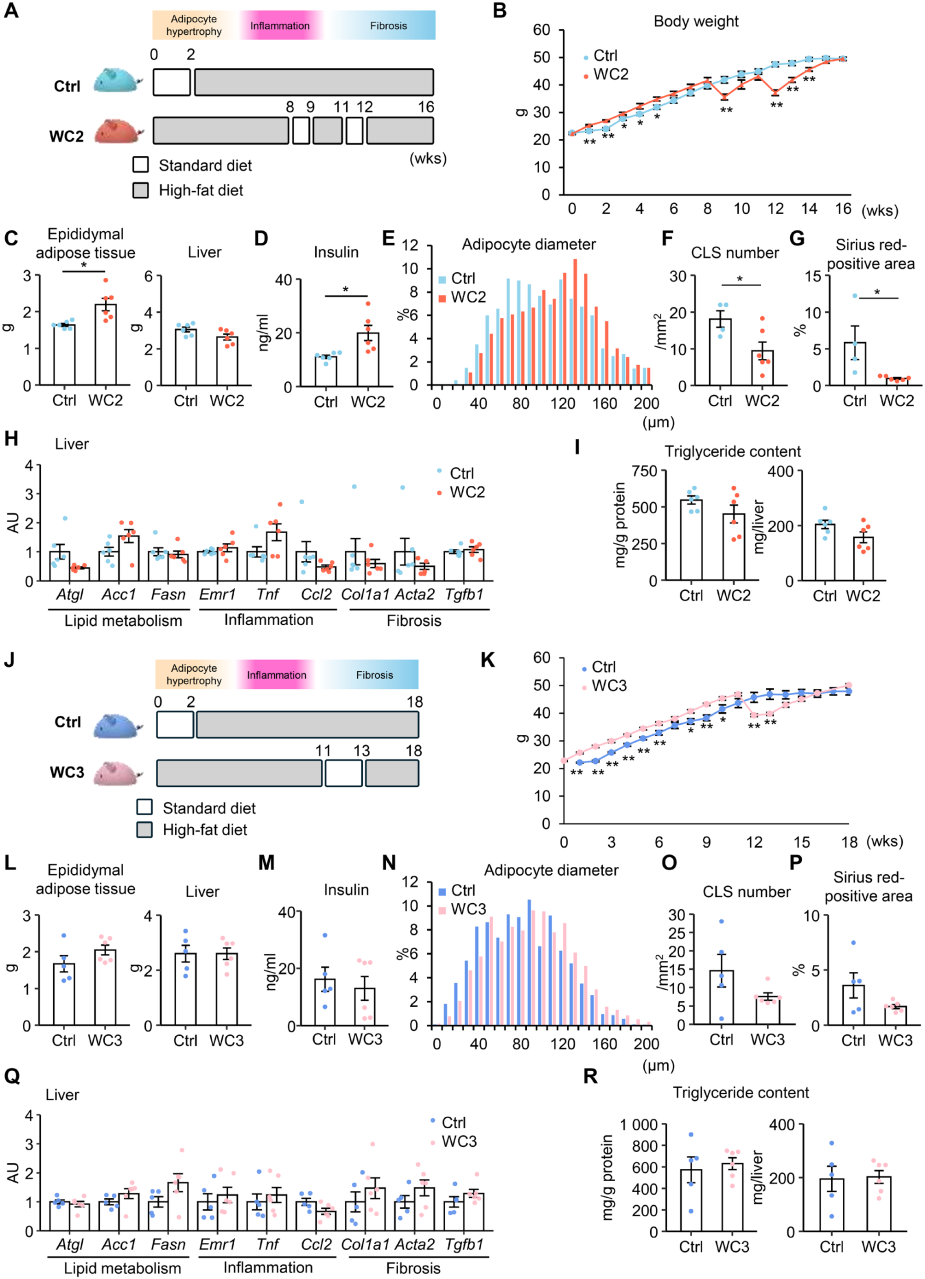
Other WC protocols show differential effects on adipose tissue. (A–I) Weight‐cycling model 2 (WC2). (A) Schematic diagram of WC2 and its control (Ctrl). The WC2 mice underwent two 1‐week periods of SD feeding with a 2‐week HFD period in between, whereas the Ctrl mice were fed HFD continuously for 14 weeks. *n* = 6 per group. (B) Body weight curves. (C) Tissue weights. (D) Serum insulin concentrations. (E) Histogram of adipocyte diameters in epididymal adipose tissue. (F) The number of crown‐like structures (CLSs) in epididymal adipose tissue. (G) Quantification of the Sirius red–positive area in epididymal adipose tissue (H) mRNA expression levels in liver. (I) Hepatic triglyceride content. (J–R) Weight‐cycling model 3 (WC3). (J) Schematic diagram of WC3 and Ctrl. After 11 weeks of HFD feeding, WC3 mice underwent a 2‐week weight loss intervention. *n* = 5–6. (K) Body weight curves. (L) Tissue weights. (M) Serum insulin concentrations. (N) Histogram of adipocyte diameters in epididymal adipose tissue. (O) The number of CLSs in epididymal adipose tissue. (P) Quantification of the Sirius red–positive area in epididymal adipose tissue (Q) mRNA expression levels in liver. (R) Hepatic triglyceride content. Data are expressed as means ± SEM. *p < 0.05 and **p < 0.01 by Student's *t* test.

Next, two different WC protocols, termed WC2 and WC3, were examined to clarify whether our results can be generalized or not. In the WC2 protocol, mice underwent two 1‐week periods of SD feeding with a 2‐week HFD period in between, whereas control mice were fed HFD continuously for 14 weeks (Figure 3A). The WC2 mice exhibited body weight reduction twice during the experimental period, and no difference was observed in body weight between the groups at the end of the experiment (Figure 3B). Trends in tissue weights and serum parameters in the WC2 protocol were essentially the same as those in the WC1 protocol (Figure 3C,D, Figure S3A,B). The expression of *Lep* was significantly increased, while the expression of inflammation‐ and fibrosis‐related genes was decreased in the WC2 protocol, resulting in adipocyte hypertrophy, decreased CLS formation, and reduced interstitial fibrosis, as observed in the WC1 protocol (Figure 3E–G, Figure S2C–E). In contrast to the WC1 protocol, mRNA expression related to lipid metabolism, inflammation, and fibrosis as well as the triglyceride content in the liver remained unchanged in the WC2 protocol (Figure 3H,I). These findings suggest that the WC2 protocol, which initiates in the inflammatory phase similar to the WC1 protocol, can induce healthy adipose tissue expansion, but is insufficient to ameliorate inflammation and fibrosis enough to reduce ectopic lipid accumulation in the liver, likely due to the short duration of weight loss.

In the WC3 protocol, mice underwent 2‐week SD feeding similar to the WC1 protocol, but weight loss commenced after 11 weeks of HFD feeding (in the fibrotic phase; Figure 3J). No statistically significant difference was observed in body, adipose tissues, or liver weights between the groups at the end of the experiment (Figure 3K,L, Figure S4A). Distinct from the WC2 protocol, the WC3 protocol did not affect adipocyte hypertrophy, CLS formation, or interstitial fibrosis, or gene expression profiles in the epididymal adipose tissue, whereas both protocols showed no appreciable changes in hepatic phenotypes (Figure 3M–R, Figure S4B–E). These findings suggest that the WC3 protocol, which initiates after the mice have progressed to the fibrotic phase, exhibits only marginal effects on adipose tissue, likely due to the delayed intervention.

### 3.4 Gene Expression Profiles in Adipose Tissue During Weight Loss

**FIGURE 4:**
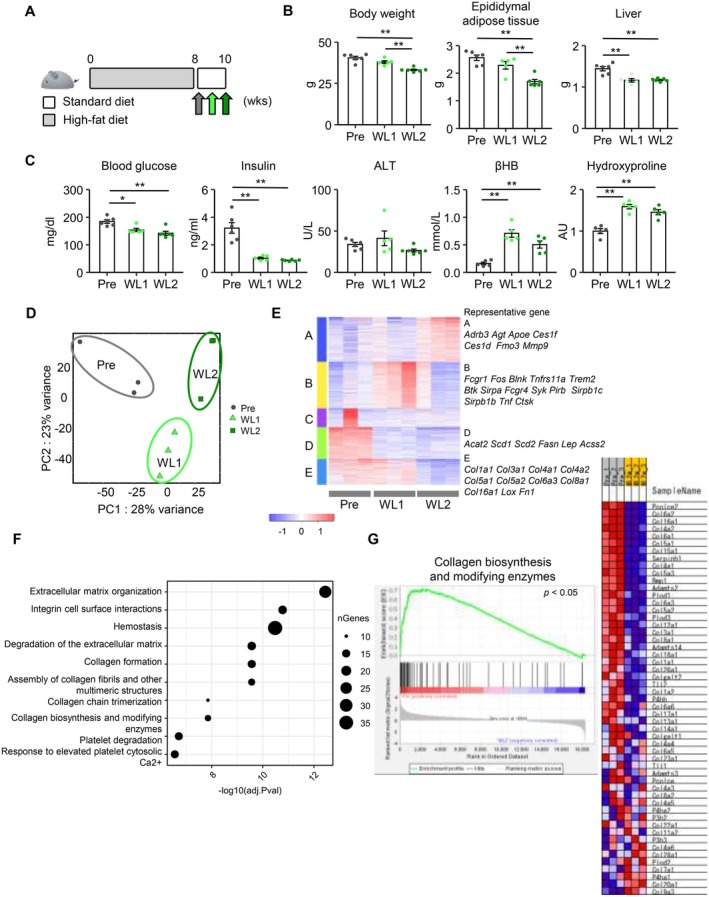
Gene expression profiles in adipose tissue during weight loss. (A) Schematic diagram of the experimental protocol. *n* = 5–6. (B) Body and tissue weights. “Pre” represents the mice fed HFD for 8 weeks. Weight loss 1 (“WL1”) and “WL2” represent the mice fed SD for 1 and 2 weeks after 8 weeks of HFD feeding, respectively. (C) Blood glucose levels and serum parameters, including β‐hydroxybutyrate (βHB) and hydroxyproline. (D–G) RNA‐sequencing analysis of epididymal adipose tissue during weight loss. *n* = 3 per group. (D) Principal component (PC) analysis and (E) K‐means clustering analyses (*k* = 5). (F) Gene enrichment analysis of cluster E. (G) Gene set enrichment analysis results of collagen biosynthesis and modifying enzymes. Data are expressed as means ± SEM. *p < 0.05 and **p < 0.01 by Tukey–Kramer test.

To elucidate the underlying mechanisms of WC‐induced healthy adipose tissue expansion, we conducted RNA‐seq analysis of epididymal adipose tissue during weight loss in the WC1 protocol (Figure 4A). As body weight decreased, the weights of epididymal adipose tissue and liver, along with serum concentrations of glucose and insulin, were reduced (Figure 4B,C). In contrast, serum concentrations of β‐hydroxybutyrate (βΗΒ), the most abundant and stable ketone body in the circulation, and hydroxyproline, a degradation product of collagens, were significantly increased throughout the weight loss period (Figure 4C). We compared gene expression profiles in epididymal adipose tissue at different phases of weight loss: before the weight loss and after 1 and 2 weeks of weight loss. Principal component analysis revealed differential gene expression profiles at each phase (Figure 4D). Using unsupervised hierarchical clustering with k‐means, five gene clusters were identified with differential expression patterns during weight loss (Figure 4E). For instance, Cluster B showed a transient inflammatory change during weight loss. Clusters D and E exhibited a reduction of mRNAs related to lipid metabolism and fibrosis after 1 and 2 weeks of weight loss, respectively (Figure 4E,F). GSEA confirmed downregulation of fibrosis‐related pathways, including collagen biosynthesis and modifying enzymes, after weight loss (Figure 4G).

### 3.5 Adipose Tissue Fibrosis Is Partially Reversed During Weight Loss

**FIGURE 5:**
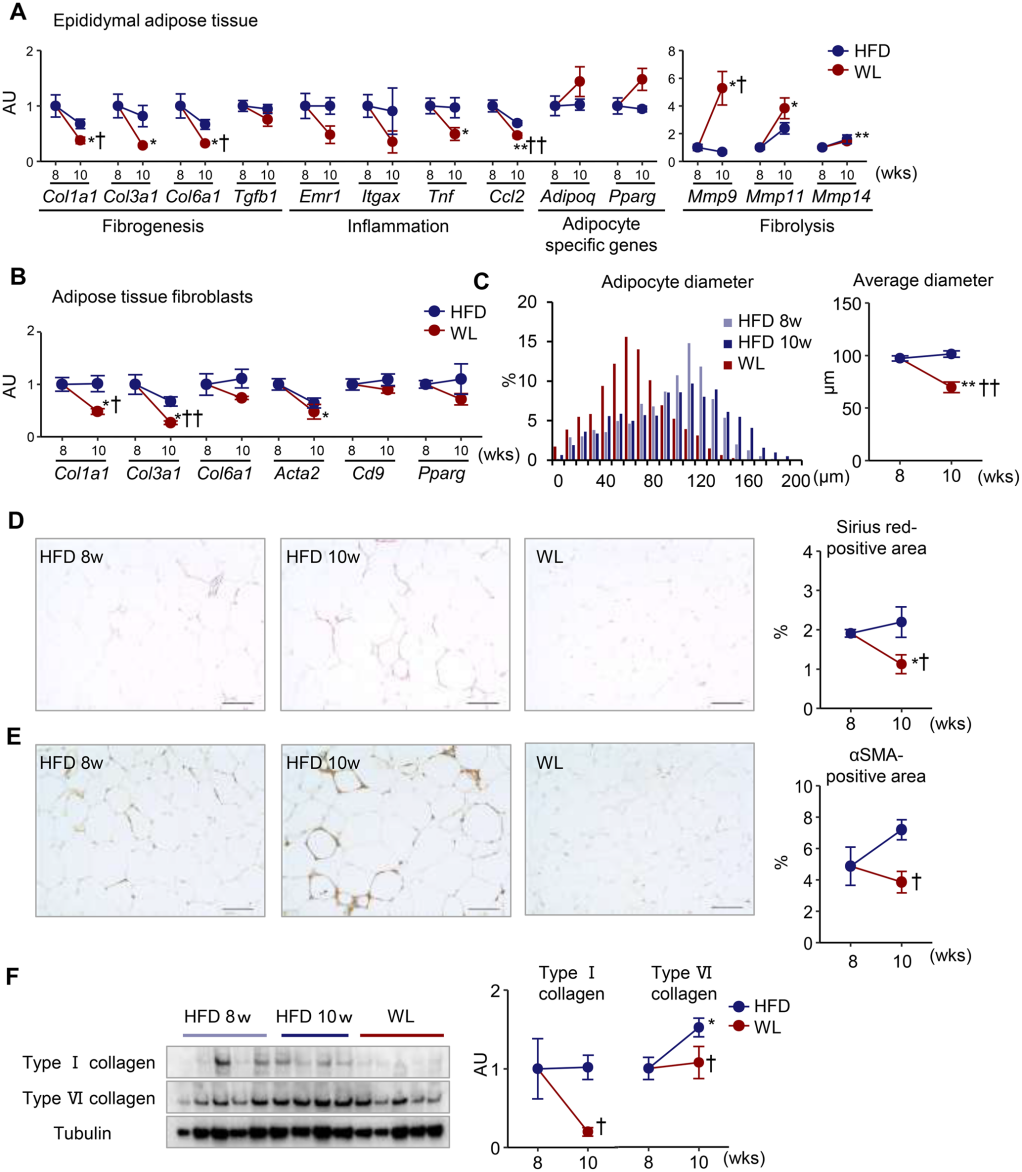
Adipose tissue fibrosis is partially reversed during weight loss. Mice were fed HFD for 8 weeks (HFD 8 w), then fed HFD or SD for an additional 2 weeks (HFD 10 w and weight loss [WL], respectively). (A) mRNA expression levels in epididymal adipose tissue. (B) mRNA expression levels in fibroblasts isolated from epididymal adipose tissue. (C) Histogram of adipocyte diameters in epididymal adipose tissue. (D, E) Representative images of Sirius red staining (D) and α‐smooth muscle actin (αSMA) immunostaining (E) in epididymal adipose tissue and their quantification. (F) Western blot analysis of type I and VI collagen in epididymal adipose tissue. Data are expressed as means ± SEM. *p < 0.05 and **p < 0.01 versus HFD 8 w; ^†^ *p* < 0.05 and ^††^ *p* < 0.01 versus HFD 10 w by Student's *t* test. *n* = 4–6. Scale bars, 100 μm.

To assess tissue remodeling during weight loss in the WC1 protocol, we validated RNA‐seq data using quantitative PCR. We confirmed that the expression of inflammation‐ and fibrosis‐related genes was generally downregulated, whereas the expression of matrix metalloproteases, such as *matrix metalloprotease (Mmp)9*, *Mmp11*, and *Mmp14*, which are associated with fibrolysis, was upregulated (Figure 5A). Since various cell types are present in adipose tissue, we isolated fibroblasts from epididymal adipose tissue before and after weight loss, and found that collagen gene expression was significantly decreased during weight loss (Figure 5B). Histologically, adipocyte hypertrophy, interstitial fibrosis, and the positive area of α‐smooth muscle actin (αSMA), a marker for active fibroblasts, were partly reversed during weight loss (Figure 5C–E). Western blot analysis confirmed reduced collagen deposition after weight loss (Figure 5F). These observations suggest dynamic reverse tissue remodeling in adipose tissue during weight loss.

### 3.6 Treatment With βΗΒ Suppresses Obesity‐Induced Adipose Tissue Fibrosis

**FIGURE 6:**
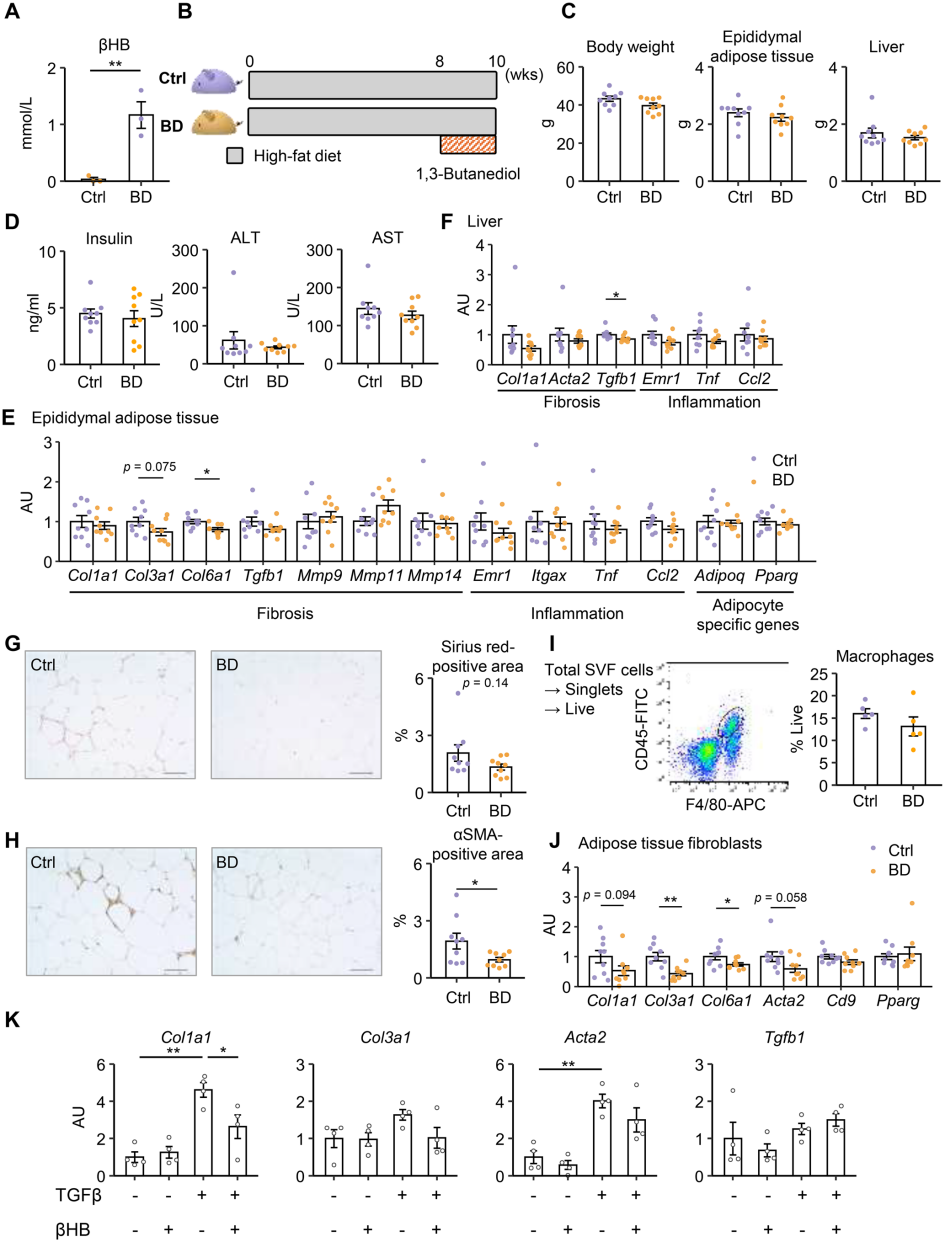
Treatment with β‐hydroxybutyrate suppresses obesity‐induced adipose tissue fibrosis. (A) Blood βHB concentrations in standard diet‐fed mice treated with 20% 1,3‐butanediol (BD) or vehicle (Ctrl). *n* = 3 per group. (B) Schematic diagram of the experimental protocol. During the last 2 weeks of a 10‐week HFD, mice were treated with BD or vehicle (Ctrl). *n* = 9 per group. (C) Body and tissue weights. (D) Serum parameters. (E) mRNA expression levels in epididymal adipose tissue. (F) mRNA expression levels in liver. (G, H) Representative images of Sirius red staining (G) and αSMA immunostaining (H) in epididymal adipose tissue and their quantification. (I) FACS gating for identification of CD45(+), F4/80 (+) (macrophages) cells in the SVF of epididymal adipose tissue (*lt*.). Proportion of macrophages in the SVF (*rt*.). *n* = 5 per group. (J) mRNA expression levels in fibroblasts isolated from epididymal adipose tissue. *n* = 9 per group. (K) Effect of βHB (5 mM) on mRNA expression levels in adipose tissue fibroblasts stimulated with TGFβ. *n* = 4. Data are expressed as means ± SEM. *p < 0.05 and **p < 0.01 by Student's *t* test (A, C–J) or analysis of variance with Tukey–Kramer test (*K*). Scale bars, 100 μm.

Considering the sustained elevation of serum βΗΒ concentration in WC1 mice during the weight loss period, but not in WC3 mice (Figure 4C, Figure S4F), we assessed the effect of βΗΒ administration on obesity‐induced adipose tissue fibrosis in mice. First, SD‐fed mice were treated with 20% BD in the drinking water, which is converted to βΗΒ by the liver [36, 37]. The blood βΗΒ levels were almost comparable to those observed during weight loss in the WC1 protocol (Figures 4C and 6A). Then, mice were treated with BD during the last 2 weeks of a 10‐week HFD feeding (Figure 6B). The treatment did not result in significant changes in body, adipose tissue, and liver weights, or serum parameters at the end of the experimental period (Figure 6C,D). Although the treatment exhibited only marginal effects on gene expression related to inflammation and fibrosis in epididymal adipose tissue and liver (Figure 6E,F), interstitial fibrosis as well as the αSMA‐positive area was reduced by the treatment (Figure 6G,H), whereas the proportion of macrophages in the SVF remained unchanged, suggesting that BD treatment showed minimal effect on macrophage infiltration (Figure 6I). Focusing on adipose tissue fibroblasts, we found that collagen gene expression was decreased in isolated fibroblasts from BD‐treated epididymal adipose tissue (Figure 6J). Moreover, βΗΒ treatment effectively suppressed the TGFβ‐stimulated increase in *Col1a1* expression in adipose tissue fibroblasts in vitro (Figure 6K), suggesting a direct effect of βΗΒ on adipose tissue fibroblasts. These results suggest that ketone bodies act on adipose tissue fibroblasts to suppress collagen production, thereby contributing, at least partly, to the amelioration of adipose tissue fibrosis during weight loss.

### 3.7 Transient BD Administration During HFD Feeding Is Sufficient to Induce Healthy Adipose Tissue Expansion

**FIGURE 7:**
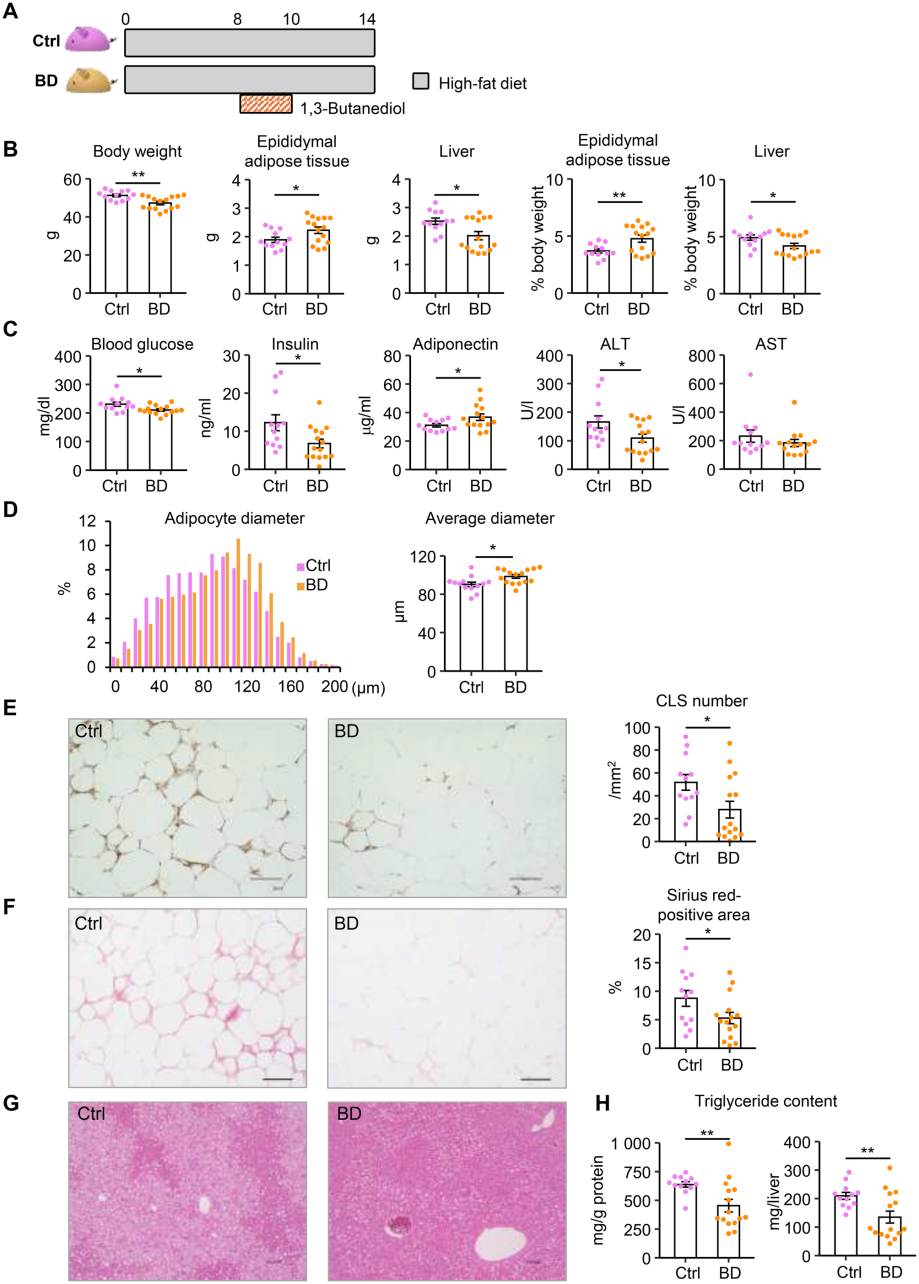
Transient BD administration during HFD feeding is sufficient to induce healthy adipose tissue expansion. (A) Schematic diagram of the experimental protocol. Mice received a transient treatment of 1,3‐butanediol (BD) for 2 weeks during a continuous 14‐week HFD feeding. *n* = 12–15. (B) Body and tissue weights, and the percentages of tissue weights relative to body weight. Ctrl, control (C) Serum parameters. (D) Histogram of adipocyte diameters in the epididymal adipose tissue. (E) Representative images of F4/80 immunostaining and the number of crown‐like structures (CLSs) in epididymal adipose tissue. (F) Representative images of Sirius red staining and quantification of the Sirius red–positive area in epididymal adipose tissue. (G) Representative images of hematoxylin and eosin staining in the liver. (H) Hepatic triglyceride content. Data are expressed as means ± SEM. *p < 0.05 and **p < 0.01 by Student's *t* test. Scale bars, 100 μm.

Our results led us to hypothesize that transient BD administration during HFD loading is sufficient to induce healthy adipose tissue expansion. To validate this hypothesis, mice were treated with BD for 2 weeks during a continuous 14‐week HFD feeding (Figure 7A). At the end of the experimental period, BD‐treated mice showed a modest decrease in body weight compared to control mice (Figure 7B). The weight of epididymal adipose tissue was significantly increased, while the liver weight was decreased in BD‐treated mice (Figure 7B). Serum parameters revealed metabolically favorable phenotypes in BD‐treated mice (Figure 7C). In epididymal adipose tissue of BD‐treated mice, the expression of *Lep* and *Adrb3* was significantly increased, while *Tgfb1* expression was reduced (Figure S5A). Histological examinations of epididymal adipose tissue revealed adipocyte hypertrophy, reduced CLS formation, and suppression of interstitial fibrosis in BD‐treated mice compared to control mice (Figure 7D–F). Moreover, hepatic expression of *Acc1* and hepatic steatosis were significantly reduced in BD‐treated mice (Figure 7G,H, Figure S5B). These phenotypes are roughly comparable to WC mice (Figures 1 and 2). Collectively, these findings indicate that BD‐induced transient hyperketonemia, when properly timed, effectively promotes healthy adipose tissue expansion with reduced ectopic lipid accumulation in diet‐induced obese mice, as observed with transient weight loss in the WC1 protocol.

## 4 Discussion

Patients with obesity often struggle to maintain dietary therapy, the cornerstone of obesity treatment, over a long period of time. In particular, it remains unclear how weight regain during obesity treatment affects systemic metabolic conditions, a phenomenon that often causes psychological stress and anxiety in patients. This study demonstrates that transient dietary intervention can induce healthy adipose tissue expansion leading to MHO, likely through the suppression of adipose tissue remodeling. Although the assessment of MHO in mouse models remains inconsistent, healthy adipose tissue expansion, reduced ectopic fat accumulation, and improved insulin resistance observed in this study are consistent with previously reported characteristics of the MHO phenotype [38]. Unlike other studies focusing on immune cells such as macrophages, this study is unique in highlighting the role of adipose tissue fibroblasts during weight loss. Although organ fibrosis has long been considered an irreversible endpoint of chronic inflammation, recent evidence reveals that it may be partially reversible, depending on the specific disease and affected organ [39, 40]. Our findings from mouse models cannot be directly translated to clinical practice in humans in their current protocol. In this regard, serum biomarkers and/or biological imaging reflecting adipose tissue fibrosis would pave the way for determining the optimal timing of weight loss intervention and treatment duration. Nevertheless, this study is timely since the development of various anti‐obesity interventions, such as glucagon‐like peptide 1 (GLP‐1) receptor agonists and related compounds, has allowed patients with obesity to reduce their body weight by over 10% [41]. Given that weight regain often occurs when such treatments are discontinued, it is crucial to elucidate the effects of WC on adipose tissue remodeling and systemic metabolic conditions, considering the types and durations of interventions.

Similar to other chronic inflammatory diseases, adipose tissue in obesity eventually develops fibrosis, leading to organ dysfunction. Over the past decade, substantial knowledge about adipose tissue fibroblasts has accumulated. For instance, PDGFRα‐positive adipocyte progenitor cells become CD9‐high and acquire profibrotic properties during the development of obesity [6]. Intermittent fasting potently suppresses cellular senescence in adipocyte progenitor cells of aging mice and restores their ability to differentiate into adipocytes [42]. Moreover, deficiency of type VI collagen in mice promotes healthy adipose tissue expansion and MHO [28]. These reports indicate the critical role of adipose tissue fibrosis in obesity‐induced metabolic derangements. However, the reversibility of adipose tissue fibrosis still remains unclear. For instance, in patients with obesity who have successfully lost weight through bariatric surgery, type I collagen synthesis in adipose tissue decreases immediately after surgery, but its degradation only increases after 1 year [43]. This suggests that reverse remodeling may be limited in patients with morbid obesity accompanied by advanced adipose tissue fibrosis. Indeed, in this study using mouse models, the intervention protocol that was initiated after adipose tissue fibrosis had progressed did not lead to significant improvement in metabolic conditions. Therefore, it is important to understand in detail how each nutritional intervention affects adipose tissue fibrosis during weight loss. There must be complex mechanisms by which transient weight loss induces healthy adipose tissue expansion, such as hypothalamic neuronal signaling, sympathetic nerve activity, and transcriptional regulation in adipocytes.

Another key finding of this study is that transient hyperketonemia can promote healthy adipose tissue expansion. Although it is known that ketone bodies administration and ketogenic diets potently induce weight loss and suppress chronic inflammation in adipose tissue, we demonstrate for the first time that transient treatment with βΗΒ, a ketone body precursor, has long‐lasting effects on adipose tissue remodeling. As the underlying mechanism, we found that ketone bodies act directly on adipose tissue fibroblasts to suppress myofibroblast transdifferentiation and collagen production. The effect of such ketone bodies may lead to reduced adipose tissue fibrosis with adipocyte hypertrophy. In this regard, Lecoutre et al. reported similar findings, highlighting the potential mechanism by which ketone bodies suppress TGFβ signaling in adipocyte progenitor cells [44]. In addition, various types of cells within adipose tissue may also be targets of ketone bodies' action. For instance, ketone bodies enhance PPARγ signaling, promote lipid synthesis, and reduce reactive oxygen species in adipocytes [45]. Ketone bodies also act on immune cells to suppress inflammation [46, 47]. In this study, BD administration did not affect macrophage infiltration or inflammatory response. Such inconsistency may be attributed to in vitro versus in vivo experimental conditions or dosages used. From the perspective of long‐lasting effects, ketone bodies–mediated epigenetic mechanisms during weight loss should be further explored [48]. Since hyperketonemia can be induced by intermittent fasting, ketogenic diets, and various medications such as sodium glucose co‐transporter 2 inhibitors [27, 49, 50, 51], it would be of value to investigate how ketone bodies regulate different cell types within adipose tissue during the development of obesity. Notably, obese individuals have reduced sensitivity to the insulin‐mediated suppression of ketogenesis, resulting in lower serum ketone body concentrations under fasted conditions [52, 53]. Therefore, exogenous administration of BD may represent a potential therapeutic strategy for obesity.

Several limitations of this study should be noted. This study focused primarily on adipose tissue and did not include a detailed evaluation of other organs, particularly skeletal muscle, where BCAA are predominantly metabolized. We did not assess the long‐term effects of WC or the effect of multiple WC interventions. In addition, it is important to conduct these experiments using female mice, given the significant clinical interest in women's weight fluctuations, particularly in relation to pregnancy and childbirth.

In conclusion, this study provides evidence that an appropriate dietary intervention can promote healthy adipose tissue expansion in mice, even after weight regain, thereby leading to MHO (Figure S6). In contrast, inappropriate protocols, such as those of short duration and late initiation, fail to induce healthy adipose tissue expansion. Regarding the underlying mechanism, our data have revealed the key role of ketone bodies, which increase in serum concentrations during weight loss. Adipose tissue fibroblasts are targets of ketone bodies, leading to the suppression of obesity‐induced adipose tissue remodeling. Beyond genetic engineering and compound administration, this study paves the way for nutritional approaches to induce MHO.

## Author Contributions

**Eri Wada:** writing – original draft, investigation, methodology, funding acquisition. **Hirotaka Hosono:** writing – original draft, investigation, methodology. **Miyako Tanaka:** writing – review and editing, formal analysis, project administration, funding acquisition. **Fumi Miyakawa**, **Hiro Kohda**, **Shogo Tanno**, **Reon Shimano:** investigation. **Kozue Ochi:** investigation, validation. **Ayaka Ito:** writing – review and editing. **Yasuyuki Kitaura**, **Kazuya Ichihara**, **Akinobu Matsumoto**, **Tomoo Ogi:** resource, methodology. **Noriko Satoh‐Asahara**, **Toyoaki Murohara:** supervision. **Takayoshi Suganami:** supervision, writing – review and editing, conceptualization, funding acquisition.

## Conflicts of Interest

The authors declare no conflicts of interest.

## Supporting information


Figure S1.

Table S1.

## Data Availability

All data generated or analyzed during this study are included in this published article and its supporting information files.
