# Supplementary material for: Transient Dietary Intervention Induces Healthy Adipose Tissue Expansion and Metabolically Healthy Obesity in Mice
Source: FASEB J. 2025 Jul 16;39(14):e70847. doi: 10.1096/fj.202501121R (PMC12265394; doi:10.1096/fj.202501121R)
Supplement: Supplementary file 1 — Figure S1. [file FSB2-39-e70847-s002.pptx]

## Slide 1
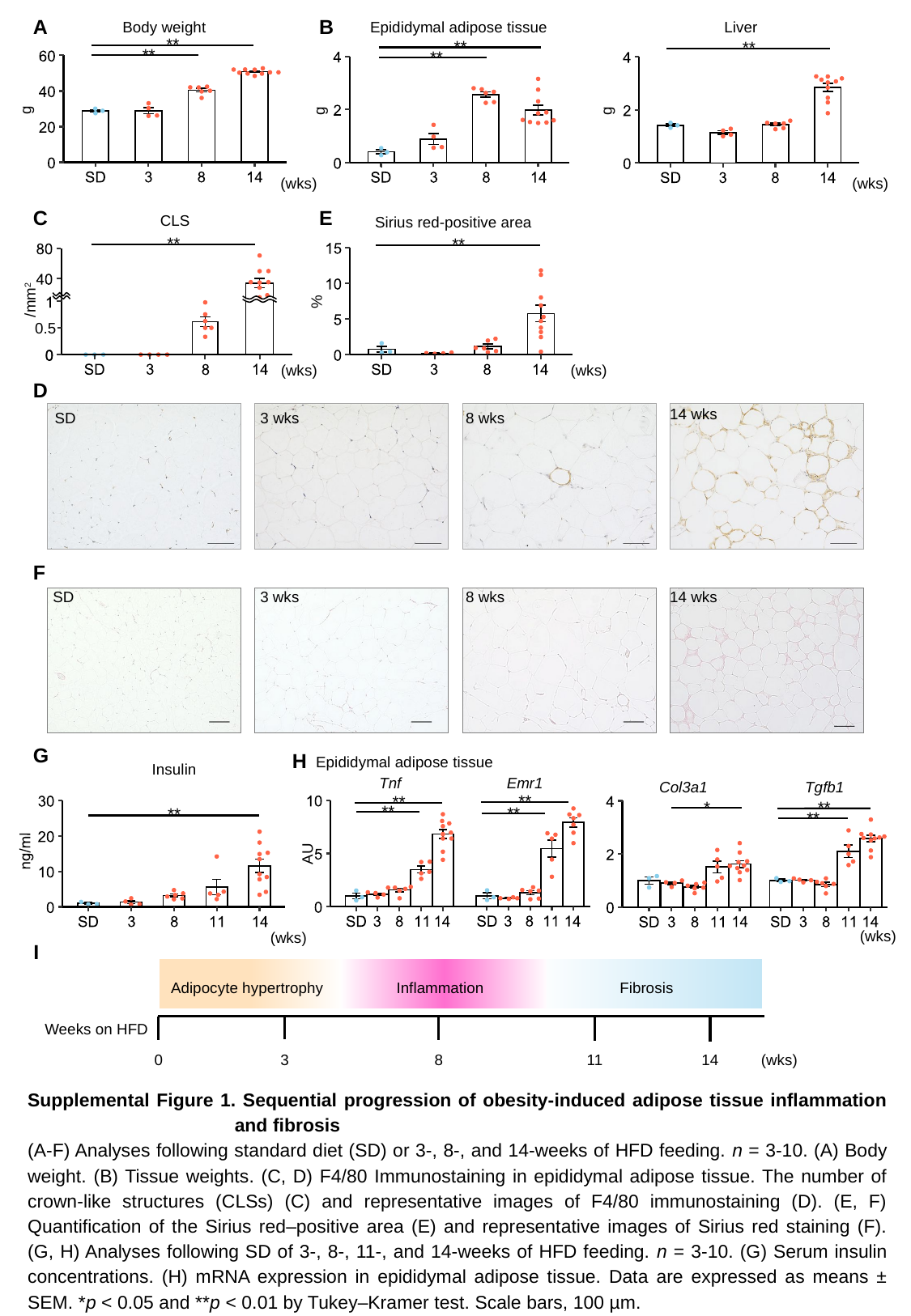

A
B
Epididymal adipose tissue
Liver
Body weight
g
**
**
**
**
**
g
g
(wks)
(wks)
C
E
CLS
Sirius red-positive area
%
**
**
0.5
/mm2
(wks)
(wks)
D
14 wks
SD
3 wks
8 wks
F
SD
3 wks
8 wks
14 wks
G
H
Epididymal adipose tissue
Insulin
Tnf
Emr1
Col3a1
Tgfb1
**
**
**
*
**
**
**
**
AU
(wks)
(wks)
I
Adipocyte hypertrophy
Inflammation
Fibrosis
Weeks on HFD
(wks)
14
0
3
8
11
Supplemental Figure 1. Sequential progression of obesity-induced adipose tissue inflammation and fibrosis
(A-F) Analyses following standard diet (SD) or 3-, 8-, and 14-weeks of HFD feeding. n = 3-10. (A) Body weight. (B) Tissue weights. (C, D) F4/80 Immunostaining in epididymal adipose tissue. The number of crown-like structures (CLSs) (C) and representative images of F4/80 immunostaining (D). (E, F) Quantification of the Sirius red–positive area (E) and representative images of Sirius red staining (F). (G, H) Analyses following SD of 3-, 8-, 11-, and 14-weeks of HFD feeding. n = 3-10. (G) Serum insulin concentrations. (H) mRNA expression in epididymal adipose tissue. Data are expressed as means ± SEM. *p < 0.05 and **p < 0.01 by Tukey–Kramer test. Scale bars, 100 µm.

## Slide 2
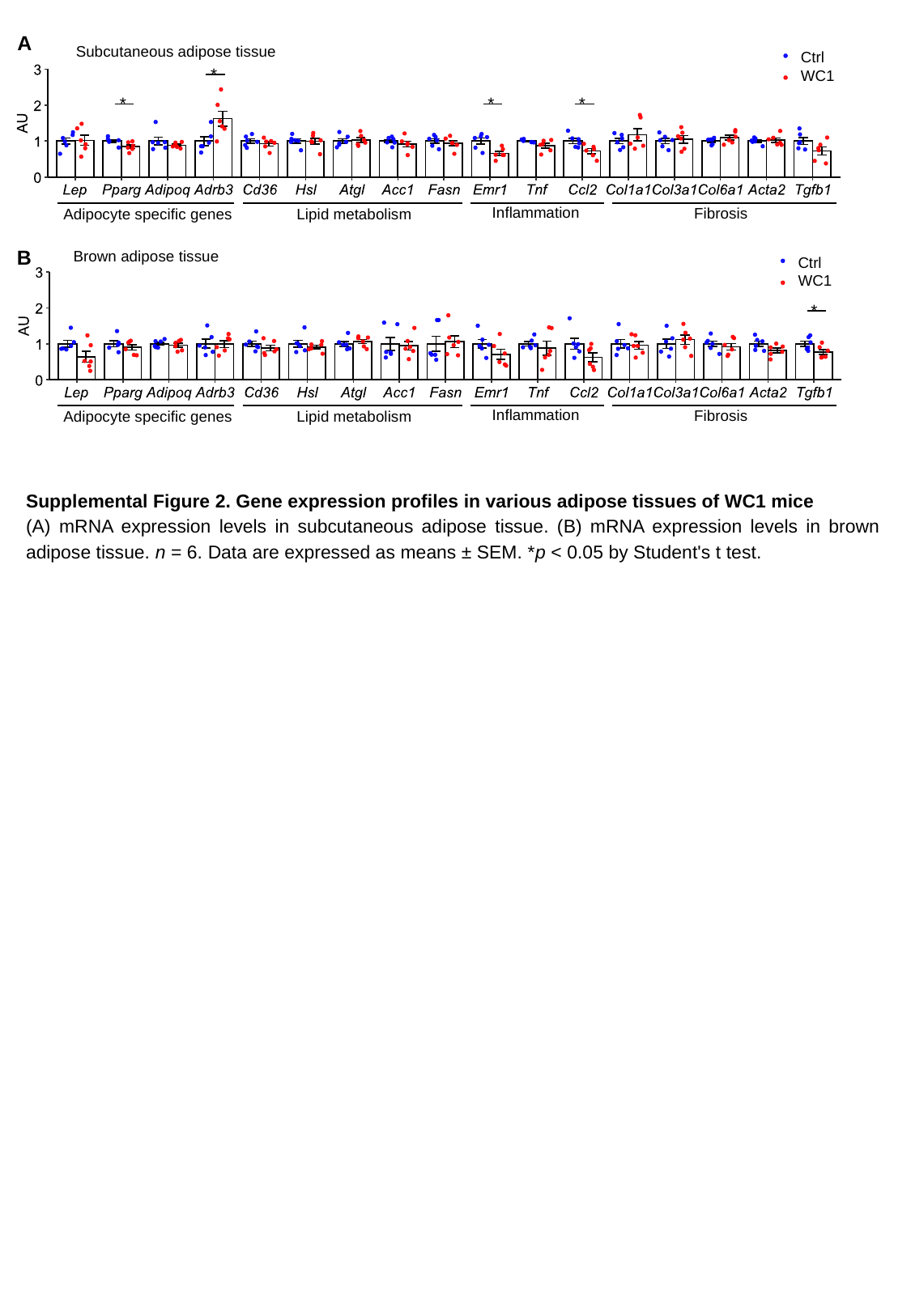

A
Subcutaneous adipose tissue
Ctrl
WC1
*
*
*
*
Inflammation
Fibrosis
Lipid metabolism
Adipocyte specific genes
B
Brown adipose tissue
Ctrl
WC1
*
Inflammation
Fibrosis
Lipid metabolism
Adipocyte specific genes
Supplemental Figure 2. Gene expression profiles in various adipose tissues of WC1 mice
(A) mRNA expression levels in subcutaneous adipose tissue. (B) mRNA expression levels in brown adipose tissue. n = 6. Data are expressed as means ± SEM. *p < 0.05 by Student's t test.

## Slide 3
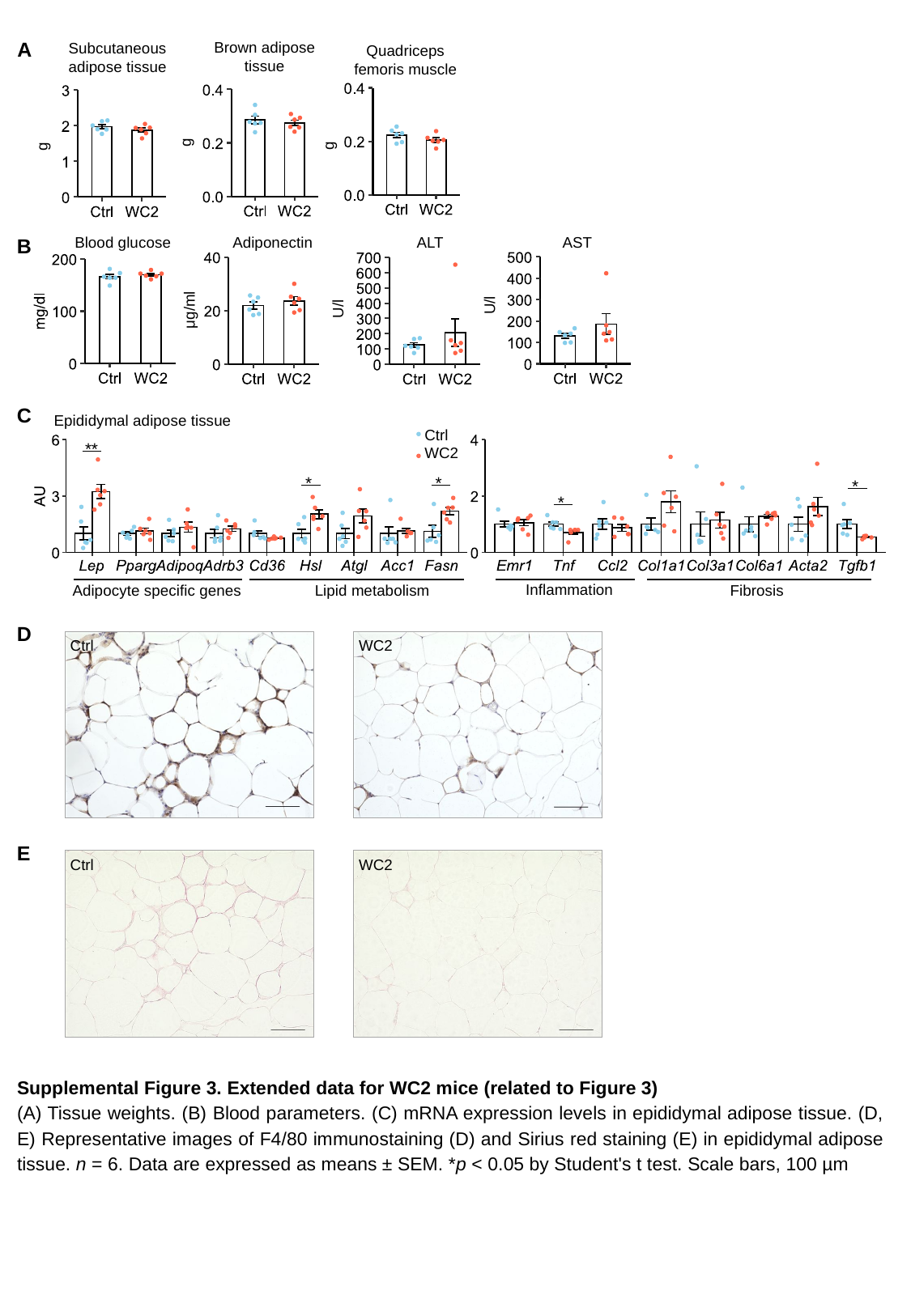

A
Brown adipose tissue
g
Subcutaneous
adipose tissue
g
Quadriceps femoris muscle
g
Blood glucose
Adiponectin
ALT
AST
B
U/l
μg/ml
U/l
C
Epididymal adipose tissue
Ctrl
WC2
**
*
*
*
*
Inflammation
Fibrosis
Lipid metabolism
Adipocyte specific genes
D
Ctrl
WC2
E
Ctrl
WC2
Supplemental Figure 3. Extended data for WC2 mice (related to Figure 3)
(A) Tissue weights. (B) Blood parameters. (C) mRNA expression levels in epididymal adipose tissue. (D, E) Representative images of F4/80 immunostaining (D) and Sirius red staining (E) in epididymal adipose tissue. n = 6. Data are expressed as means ± SEM. *p < 0.05 by Student's t test. Scale bars, 100 µm

## Slide 4
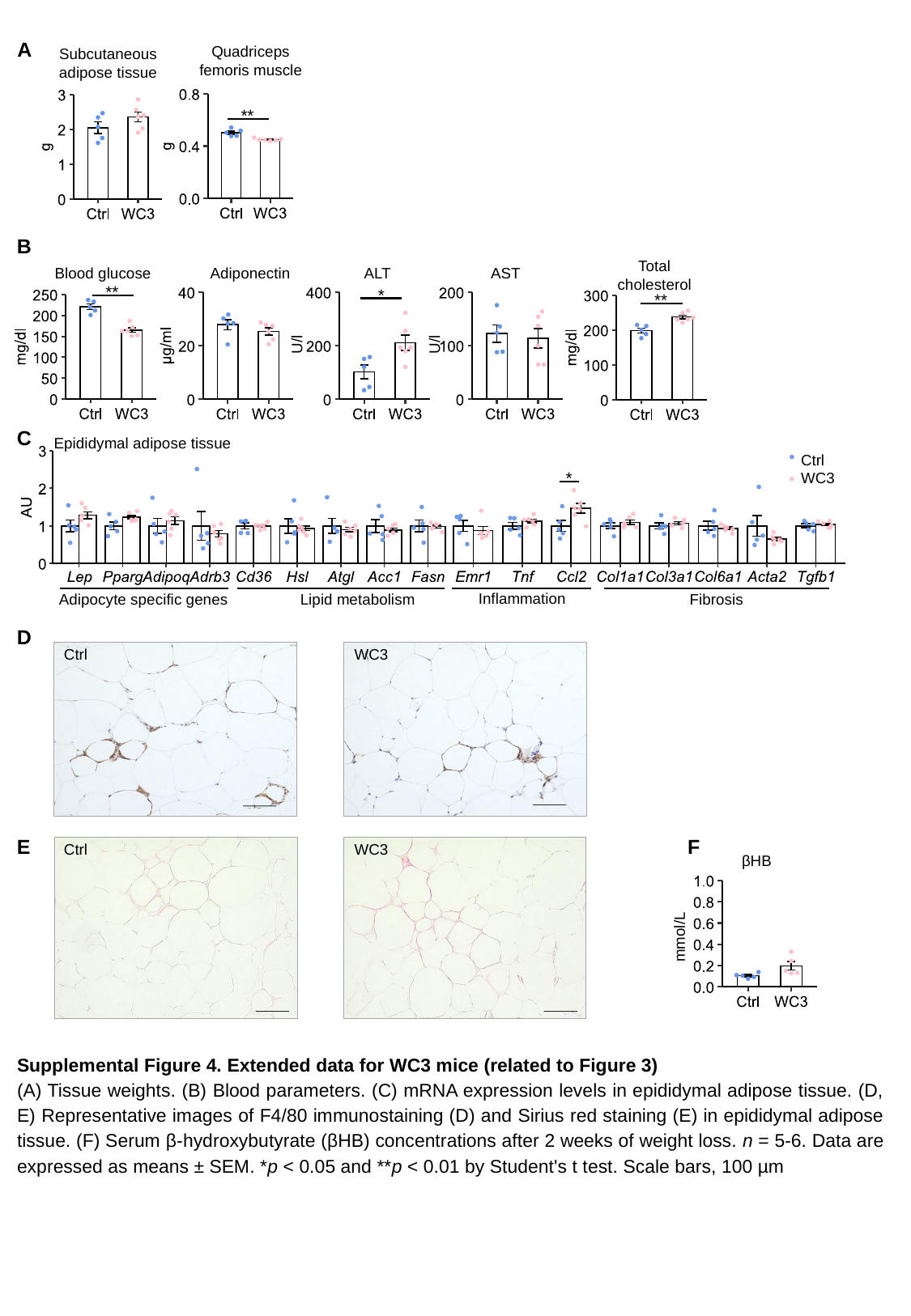

A
Quadriceps femoris muscle
Subcutaneous
adipose tissue
**
B
Total cholesterol
Blood glucose
Adiponectin
ALT
AST
**
*
**
U/l
U/l
C
Epididymal adipose tissue
Ctrl
WC3
*
Inflammation
Fibrosis
Lipid metabolism
Adipocyte specific genes
D
Ctrl
WC3
E
F
Ctrl
WC3
βHB
mmol/L
Supplemental Figure 4. Extended data for WC3 mice (related to Figure 3)
(A) Tissue weights. (B) Blood parameters. (C) mRNA expression levels in epididymal adipose tissue. (D, E) Representative images of F4/80 immunostaining (D) and Sirius red staining (E) in epididymal adipose tissue. (F) Serum β-hydroxybutyrate (βHB) concentrations after 2 weeks of weight loss. n = 5-6. Data are expressed as means ± SEM. *p < 0.05 and **p < 0.01 by Student's t test. Scale bars, 100 µm

## Slide 5
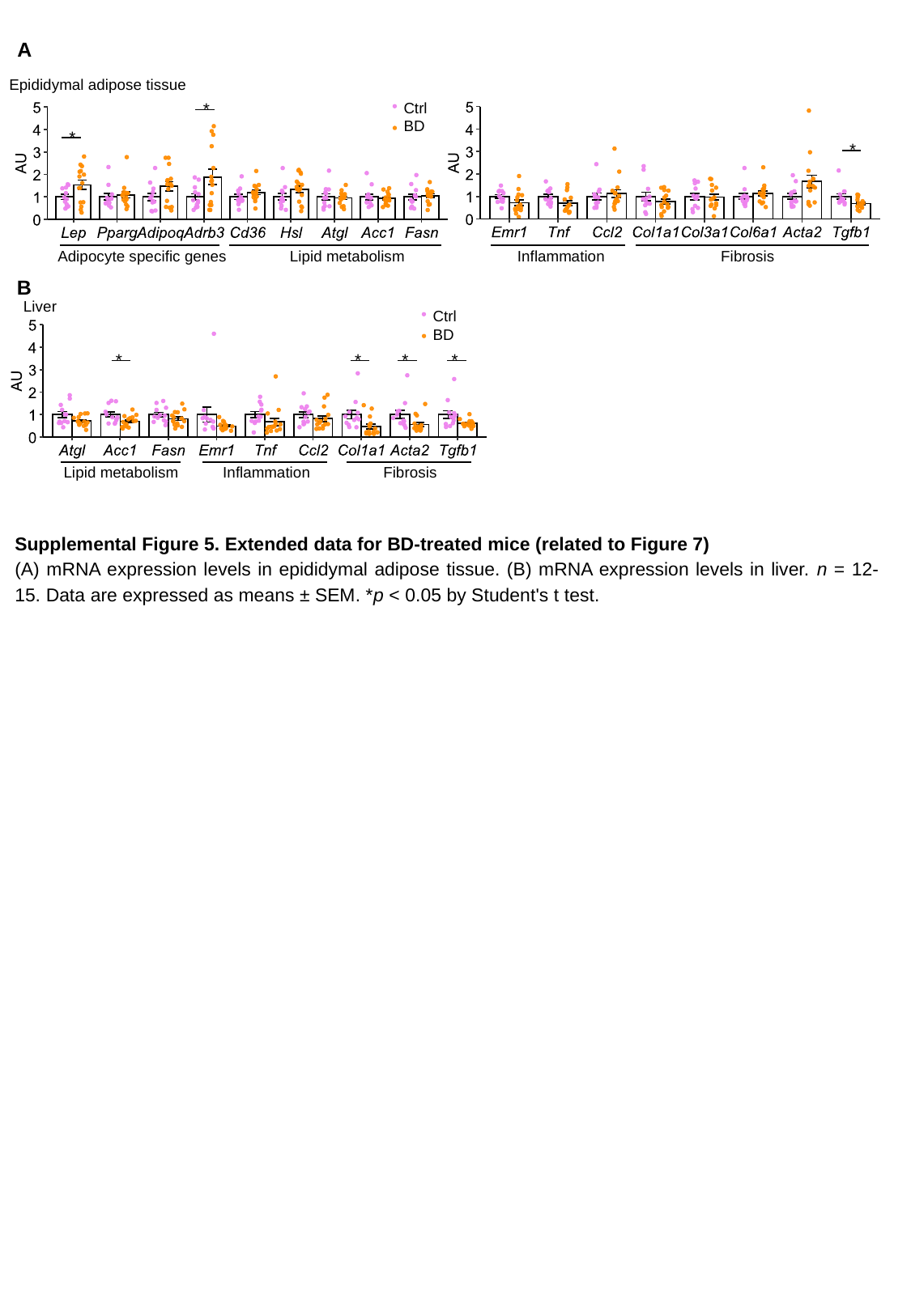

A
Epididymal adipose tissue
*
Ctrl
BD
*
*
Adipocyte specific genes
Lipid metabolism
Inflammation
Fibrosis
B
Liver
Ctrl
BD
*
*
*
*
Inflammation
Fibrosis
Lipid metabolism
Supplemental Figure 5. Extended data for BD-treated mice (related to Figure 7)
(A) mRNA expression levels in epididymal adipose tissue. (B) mRNA expression levels in liver. n = 12-15. Data are expressed as means ± SEM. *p < 0.05 by Student's t test.

## Slide 6
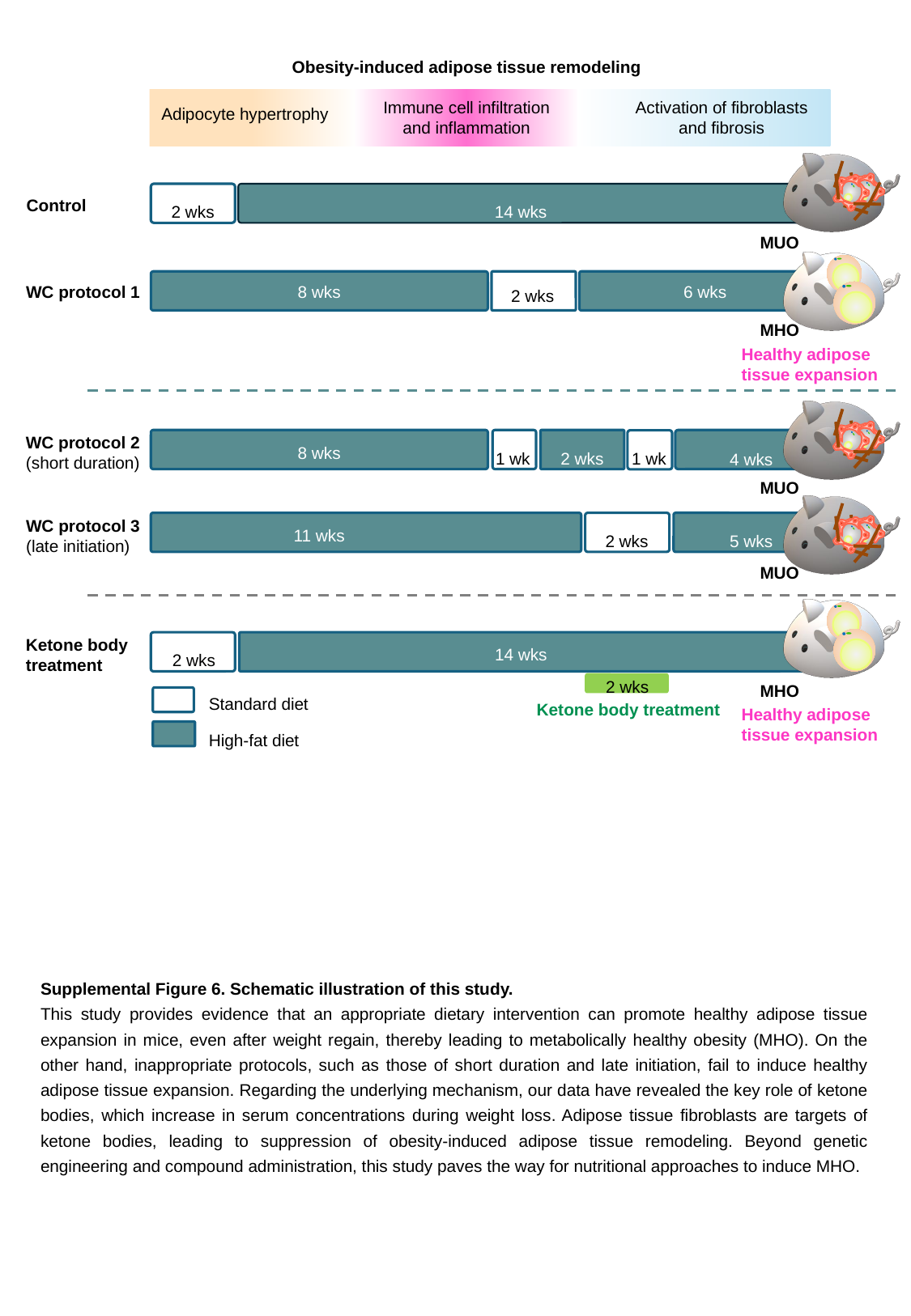

Obesity-induced adipose tissue remodeling
Immune cell infiltration
and inflammation
Activation of fibroblasts and fibrosis
Adipocyte hypertrophy
2 wks
14 wks
Control
MUO
2 wks
WC protocol 1
8 wks
6 wks
MHO
Healthy adipose
tissue expansion
WC protocol 2
(short duration)
1 wk
2 wks
1 wk
4 wks
8 wks
MUO
WC protocol 3
(late initiation)
2 wks
5 wks
11 wks
MUO
Ketone body treatment
2 wks
14 wks
2 wks
MHO
Standard diet
Ketone body treatment
Healthy adipose
tissue expansion
High-fat diet
Supplemental Figure 6. Schematic illustration of this study.
This study provides evidence that an appropriate dietary intervention can promote healthy adipose tissue expansion in mice, even after weight regain, thereby leading to metabolically healthy obesity (MHO). On the other hand, inappropriate protocols, such as those of short duration and late initiation, fail to induce healthy adipose tissue expansion. Regarding the underlying mechanism, our data have revealed the key role of ketone bodies, which increase in serum concentrations during weight loss. Adipose tissue fibroblasts are targets of ketone bodies, leading to suppression of obesity-induced adipose tissue remodeling. Beyond genetic engineering and compound administration, this study paves the way for nutritional approaches to induce MHO.
