# Supplementary material for: Transient Dietary Intervention Induces Healthy Adipose Tissue Expansion and Metabolically Healthy Obesity in Mice
Source: FASEB J. 2025 Jul 16;39(14):e70847. doi: 10.1096/fj.202501121R (PMC12265394; doi:10.1096/fj.202501121R)
Supplement: Supplementary file 2 — Table S1. [file FSB2-39-e70847-s001.pdf]

Supplemental Table 1. Antibodies used in this study

Primary antibodies		Source	Cat#	Concentration
F4/80 antibody	rat monoclonal	Bio-Rad	MCA497	1:1000
Alpha smooth muscle Actin (αSMA) antibody	rabbit polyclonal	Abcam	ab5694	1:400
Type I Collagen antibody	goat polyclonal	Southern Biotech	1310-01	1:400
Type VI Collagen antibody	rabbit polyclonal	Abcam	ab6588	1:400
α-Tubulin antibody	mouse monoclonal	Cell Signaling Technology	3873	1:2000

Secondary antibodies	Source	Cat#	Concentration
Histofine Simple Stain Mouse MAX-PO(Rat)	NICHIREI BIOSCIENCES	414311	
Histofine Simple Stain Mouse MAX-PO(G)	NICHIREI BIOSCIENCES	414351	
Histofine Simple Stain Mouse MAX-PO(R)	NICHIREI BIOSCIENCES	414341	
Anti-Rabbit IgG, HRP-Linked Antibody	Cell Signaling Technology	7074	1:2000
Anti-mouse IgG HRP-Linked Antibody	Cell Signaling Technology	7076	1:2000

Anibodies used in flow cytometric analyses	Source	Cat#	Concentration
PE anti-mouse CD140a Antibody	BioLegend	135906	1:100
Biotin anti-mouse CD31 Antibody	BioLegend	102503	1:100
FITC anti-mouse CD45 Antibody	BioLegend	103108	1:100
APC/Cyanine7 anti-mouse CD45 Antibody	BioLegend	103116	1:100
Biotin anti-mouse CD45 Antibody	BioLegend	103103	1:100
Biotin anti-mouse TER-119/Erythroid Cells Antibody	BioLegend	116203	1:100
APC anti-mouse F4/80 Antibody	BioLegend	123116	1:100

Supplemental Table 2. Primers used in this study

Acc1	Fw	TGAGATTGGCATGGTAGCCTG
	Rv	CTCGGCCATCTGGATATTCAG
Acta2	Fw	GTTCAGTGGTGCCTCTGTCA
	Rv	ACTGGGACGACA TGGAAAAG
Adipoq	Fw	ATGGCAGAGATGGCACTCCT
	Rv	CCTTCAGCTCCTGTCATTCCA
Adrb3	Fw	ACTGCTAGCATCGAGACCTTG
	Rv	AAGGGTTGGTGACAGCTAGG
Atgl	Fw	GGTGACCATCTGCCTTCCAG
	Rv	TGCAGAAGAGACCCAGCAGT
Ccl2	Fw	CCACTCACCTGCTGCTACTCAT
	Rv	TGGTGATCCTCTTGTAGCTCTCC
Cd36	Fw	CCAAATGAAGATGAGCATAGGACA
	Rv	TCCAGTTATGGGTTCCACATCTAAG
Cd9	Fw	CCACGCAACTCCAGCTTGTAC
	Rv	GCTCTTGGTCTGAGAGTCGAATC
Col1a1	Fw	CCTCAGGGTATTGCTGGACAAC
	Rv	ACCACTTGATCCAGAAGGACCTT
Col3a1	Fw	ATGCCCACAGCCTTCTACAC
	Rv	ACCAGTTGGACATGATTCACAG
Col6a1	Fw	TGGAGCCACGTCTAAGTATCATTG
	Rv	AATTGCGCCGGTATGTGTG
Emr1	Fw	CTTTGGCTATGGGCTTCCAGTC
	Rv	GCAAGGAGGACAGAGTTTATCGTG
Fasn	Fw	CCTGGATAGCATTCCGAACCT
	Rv	AGCACATCTCGAAGGCTACACA
Hsl	Fw	GGCTTACTGGGCACAGATACCT
	Rv	CTGAAGGCTCTGAGTTGCTCAA
Itgax	Fw	GCCATTGAGGGCACAGAGA
	Rv	GAAGCCCTCCTGGGACATCT
Leptin	Fw	GAGACCCCTGTGTCGGTTC
	Rv	CTGCGTGTGTGAAATGTCATTG
Mmp9	Fw	AGGGGCGTGTCTGGAGATTC
	Rv	CCAGGGCACACCAGAGAACT
Mmp 11	Fw	CCGCCAGATGTCTGTGA
	Rv	CGCAGCCTCCACACAA
Mmp 14	Fw	GGATACCCAATGCCCATTGGCCA
	Rv	CCATTGGGCATCCAGAAGAGAGC
Pparg	Fw	ACAATGCCATCAGGTTTGGG
	Rv	CCGCCAACAGCTTCTCCTT
Tgfb1	Fw	CCTGAGTGGCTGTCTTTTGACG
	Rv	AGTGAGCGCTGAATCGAAAGC
Timp1	Fw	ATCTGGCATCCTCTTGTTG
	Rv	CGCTGGTATAAGGTGGTCTC
Tnf	Fw	ACCCTCACACTCAGATCATCTTC
	Rv	TGGTGGTTTGCTACGACGT
36B4	Fw	GGCCCTGCACTCTCGCTTTC
	Rv	TGCCAGGACGCGCTTGT
